# Supplementary material for: Peripheral Nerve Magnetoneurography With Optically Pumped Magnetometers
Source: Front Physiol. 2022 Mar 18;13:798376. doi: 10.3389/fphys.2022.798376 (PMC8975546; doi:10.3389/fphys.2022.798376)
Supplement: Supplementary file 1 [file Data_Sheet_1.pdf]

Supplementary Material

1 Supplemental Figures

1.1 Helmholtz Coils setup.

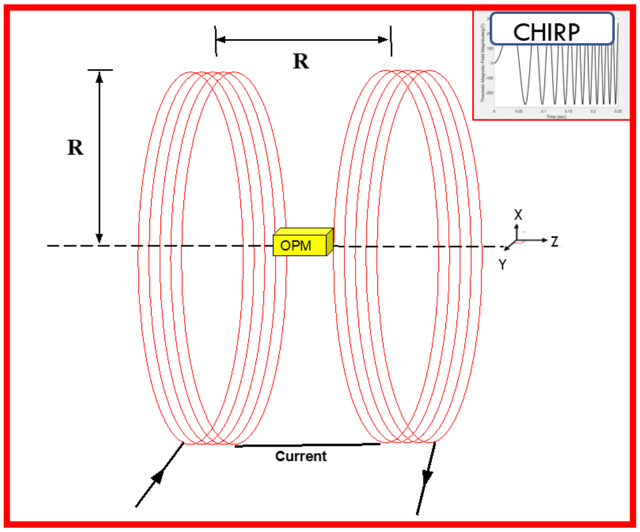

Supplemental Figure 1: OPM frequency response and sensitivity characterization setup. A single Gen-2 OPM was placed between two copper wire Helmholtz Coils (coiled red lines are 7.5 cm radius loops with 5 coils per loop) at 7.5 cm separation. Based on electromagnetic theory, the amplitude of the magnetic field in the direction of the z-axis at the center of the two coils, is given by the equation: $B_{z-axis} = \frac{\left(\frac{5}{4}\right)^{-\frac{3}{2}}\mu_0 \cdot N \cdot i}{r}$, where r is the radius of the coils, μ_0 is the relative permittivity of air, N is the number of wire loops in each coil, and i is the current running through the coils. Once the response of the OPM to several chirp functions (N=11) was recorded, the frequency response of the sensor was calculated by performing a fast Fourier transform of sensor response to each chirp and averaging each response to reduce the effects of noise. Then this Fourier transform was normalized to frequency content from the input voltage chirp. This process can be summarized mathematically with the equation: $H(jw) = \frac{\bar{x}[\mathcal{F}\{y(t)\}]}{\bar{x}[\mathcal{F}\{x(t)\}]}$, where $H(jw)$ is the frequency response, $y(t)$ is the signal recorded by the OPM, and $x(t)$ is the voltage chirp function, $\bar{x}$ denotes calculation of the mean, and $\mathcal{F}\{y(t)\}$ is the Fourier transform computed using the Fast Fourier transform algorithm. The OPM response at 5 Hz, was well within the manufactures listed bandpass of 3-125 Hz, was set to unity to create the final frequency response curve.

1.2 Surface electrode SNAP measurement

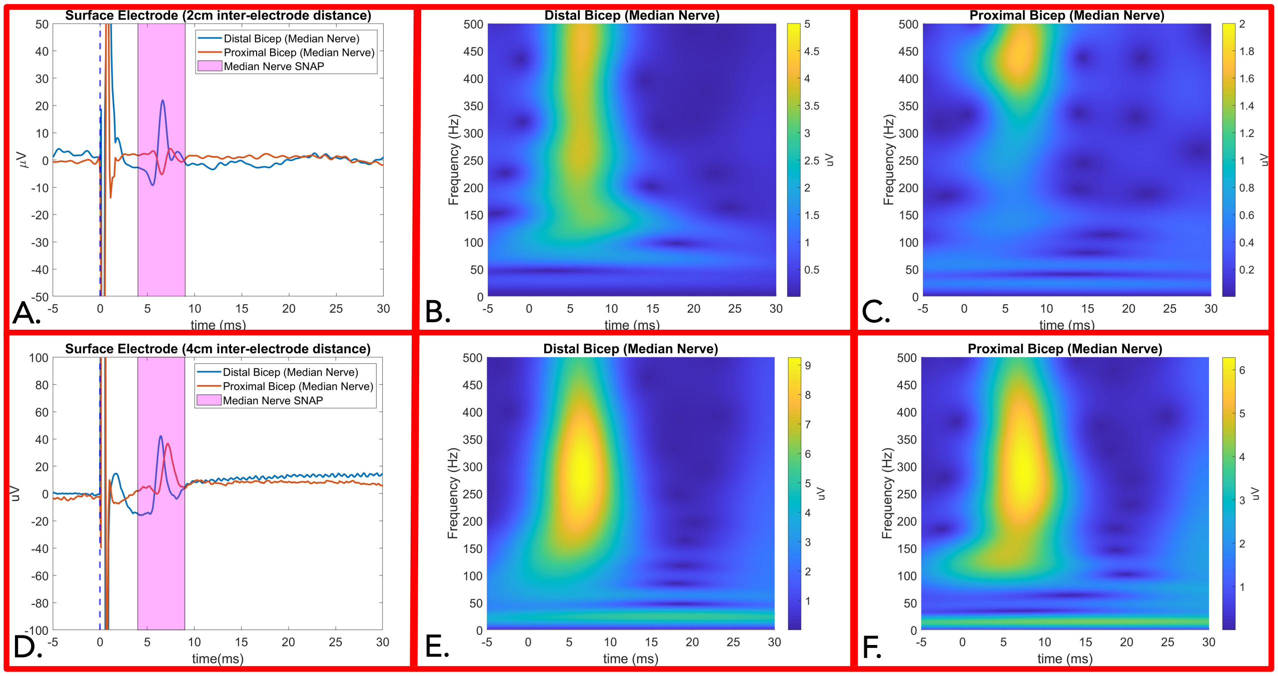

Supplemental Figure 2: Panel A and D: Time-locked average comparison from surface electrode with 2 cm **(A)** and 4 cm **(D)** inter-electrode distance for median nerve SNAP measurement demonstrated 0.8 ms temporal dispersion. SNAP action potential is marked in the magenta shaded area. **Panel B and C**: 2cm inter-electrode distance time-frequency analysis for SNAP measured at distal bicep **(B)** and proximal bicep **(C)**. Distal bicep (**B**) and proximal bicep (**C**) demonstrate equal center frequencies of 460 Hz. Distal bicep residual low frequency components (**C**) are due to triphasic action potential measured from the superficial median nerve. **Panel E and F**: 4cm inter-electrode distance time-frequency analysis for SNAP measured at distal bicep **(E)** and proximal bicep **(F)**. Distal bicep (**E**) and proximal bicep (**F**) demonstrate equal center frequencies of 300 Hz. (SNAP= Sensory Nerve Action Potential, μV = microvolt, ms=milliseconds)

1.3 OPM SNAP Raw Measurement

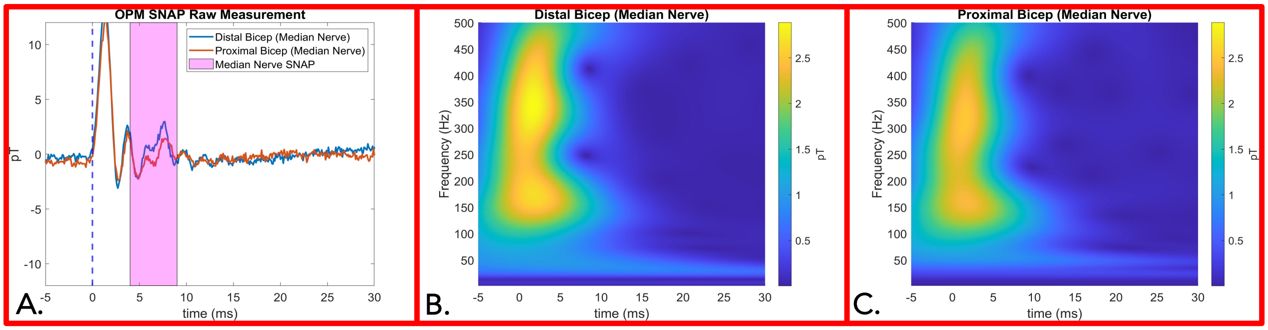

Supplemental Figure 3: **Panel A**: OPM SNAP raw measurements from median nerve at distal and proximal bicep. The OPM internal hardware digital filter at 500 Hz generates up to a 15 ms ring effect post stimulation. **Panel B and C**: Time-frequency analysis for SNAP measured by OPM before artifact removal at distal bicep **(B)** and proximal bicep **(C).** The stimulation artifact and its ringing effect distort accurate signal acquisition of the SNAP center frequency. To reduce ringing effect data contamination, both stimulation artifact and subsequent ringing effect curves were regressed as a *sinc* function by non-linear least squares method and subtracted from the averaged data. Finally, a second order 20-500 Hz bandpass filtered was applied effectively removing stimulation artifact ring effects **presented in Figure 2F.** (OPM = Optically Pumped Magnetometers, SNAP= Sensory Nerve Action Potential, pT=picoTesla, ms=milliseconds)

1.4 Ultrasound Neural Imaging Measurements

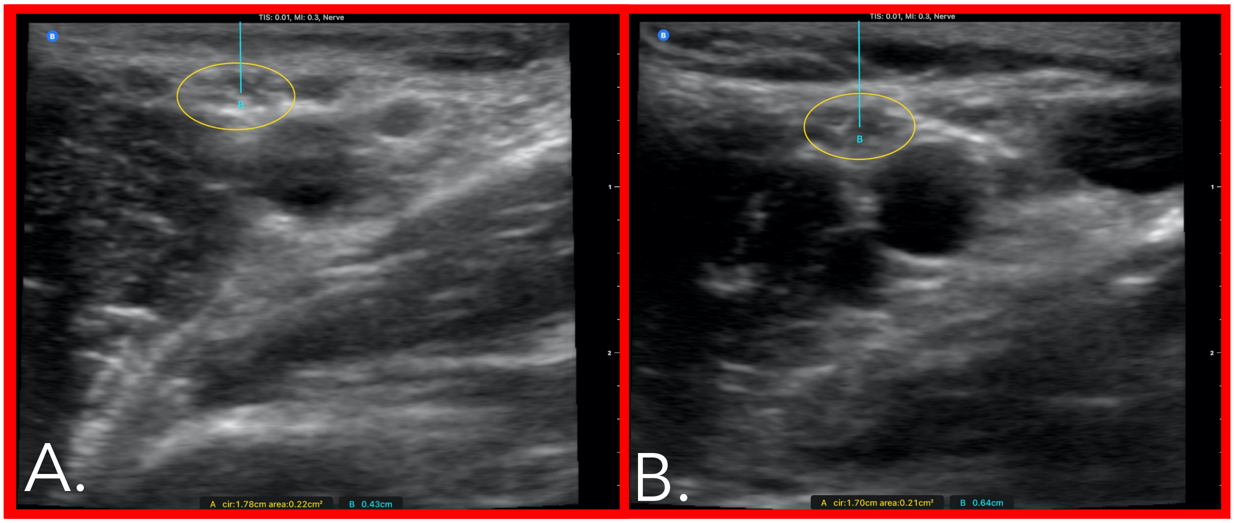

Supplemental Figure 4: Using high resolution B-mode ultrasound imaging, we accurately measured the median nerve circumference, area, and depth at the distal (**Panel A)** and proximal (**Panel B)** bicep. The median nerve at two measurement sites was similar in circumference (1.72 cm and 1.7 cm, respectively) and area (0.21 cm^2 for both) (**Panel A & B**). Compared to the median nerve localized at the distal bicep (0.43 cm) (**Panel A**), it was localized deeper at the proximal bicep (0.64 cm) (**Panel B**).

2 Supplemental Table

Supplemental Table 1: Onset latency statistic comparison for M-wave and H-Reflex measurements between Surface electrode and OPM from two subjects.

	Surface M-Wave	OPM M-Wave	Surface H-Reflex	OPM H-Reflex
Subject1	4.36 ± 0.11 ms	4.36 ± 0.24 ms	17.68 ± 0.30 ms	17.82 ± 0.37 ms
Subject2	3.06 ± 0.13 ms	3.13 ± 0.15 ms	17.52 ± 0.18 ms	17.6 ± 0.4 ms
